# Exploring “Intoxicated Syndrome”: A rare case of cervical kyphoscoliosis due to drug abuse

**DOI:** 10.1002/ccr3.9531

**Published:** 2025-04-22

**Authors:** Majid Rezvani, Seyedali Modarres Sadeghi, Farid Masaeli, Anish Thapa, Ashani Shah, Farhad Mahmoudi

**Affiliations:** ^1^ Department of Neurosurgery Isfahan University of Medical Sciences Isfahan Iran; ^2^ Universal College of Medical Sciences Bhairahawa Nepal; ^3^ Rutgers New Jersey Medical School Newark New Jersey USA; ^4^ Department of Neurology University of Miami Miami Florida USA

**Keywords:** addiction, DHS, dropped Head Syndrome, facetectomy, kyphoscoliosis

## Abstract

Dropped head syndrome (DHS), marked by severe cervical muscle weakness, causes progressive kyphosis and difficulty in maintaining head posture. This case study reports on a 23‐year‐old male with DHS linked to drug abuse, underscoring the need to consider substance abuse as a cause and highlighting effective surgical treatment.

## 1 INTRODUCTION

The cervical spine is a crucial anatomical structure that protects neurologic elements and is fundamental for preserving horizontal gaze. Maintaining the normal alignment of the cervical spine, particularly in the sagittal plane, is essential for proper physiologic functioning and minimizing muscle energy expenditure.^1^

Cervical kyphosis is the most prevalent deformity impacting the cervical spine, disrupting its physiology and resulting in considerable disability for the affected individual. This deformity may manifest as either regional or global, and multiple studies have shown its correlation with a diminished quality of life.^2^

Dropped Head Syndrome (DHS), also known as floppy head syndrome, is a rare medical disorder characterized by weakness in the neck extensor muscles, rendering them unable to support the head against gravity. This leads to a passively correctable chin‐on‐chest deformity.^3^ DHS is most commonly associated with various neuromuscular conditions, including mitochondrial myopathy, congenital myopathy, myasthenia gravis, motor neuron disease, chronic inflammatory demyelinating polyneuropathy (CIDP), and cervical myelopathy.^4^ In this study, we present an intriguing and previously undocumented case of DHS. Unlike commonly reported causes of DHS, such as neuromuscular disorders or structural abnormalities, our case diverges from these conventional etiologies. This unique presentation challenges existing paradigms and underscores the importance of further investigation into less conventional pathways leading to DHS. By thoroughly examining the patient's medical history, clinical presentation, and diagnostic findings, we aim to contribute insights that broaden our understanding of the etiologic spectrum of DHS.

## 2 CASE HISTORY/EXAMINATION

**FIGURE 1:**
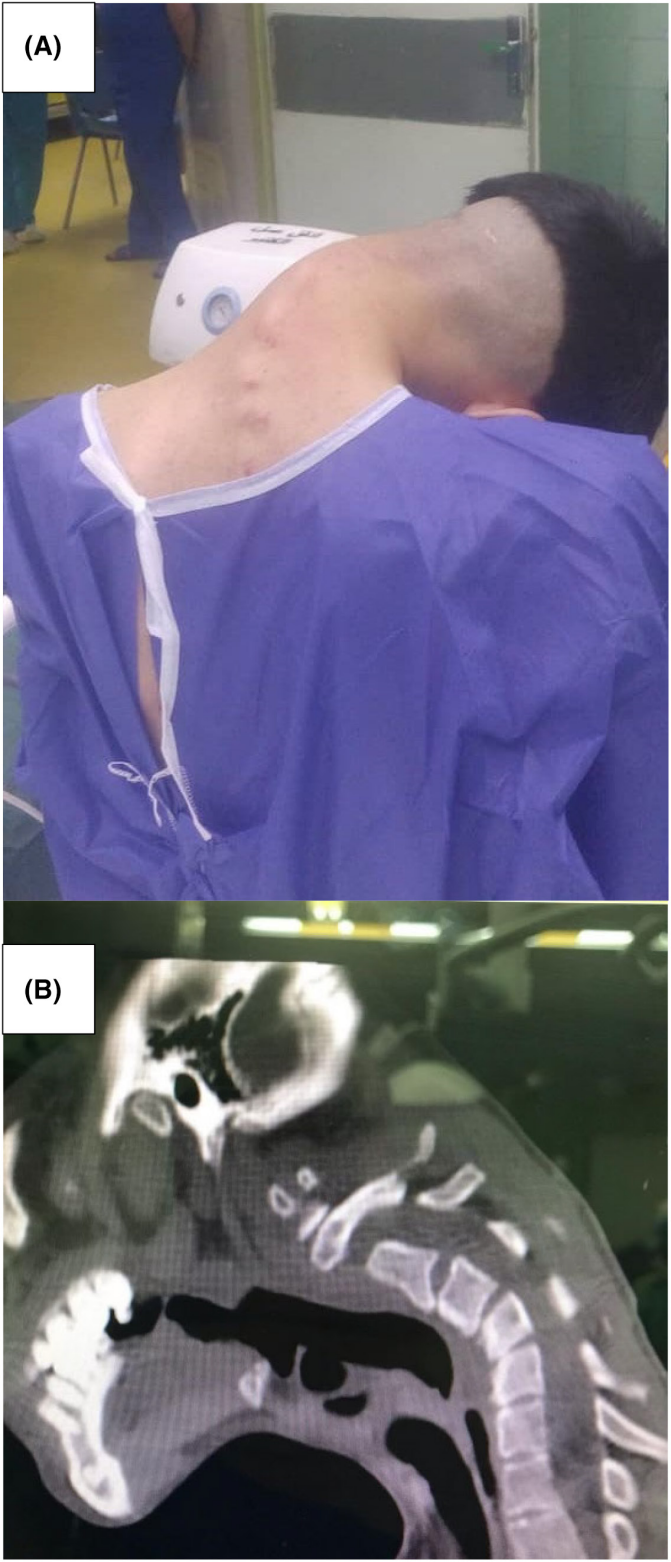
(A) Progressive cervical spine deformity in a 23‐year‐old previously healthy male due to substance abuse. (B) The CT scan of the patient reveals severe neck kyphoscoliosis.

A 23‐year‐old male with a progressive cervical spine deformity and dropped head, ongoing for the past 15 months, presented to the neurosurgery outpatient clinic at Alzahra hospital in Isfahan, Iran. The patient is experiencing chronic neck pain and upper limb paresthesia. He appears to have a slight build and exhibits a pronounced forward‐bending head posture. Additionally, there is markedly restricted range of motion (ROM) in the cervical spine, with the chin nearly in contact with the sternum manubrium. He has no history of severe neck trauma or neck surgery, and the kyphosis is not passively correctible. The patient, hailing from a socioeconomically disadvantaged background, has a significant medical history of major depressive disorder and substance abuse, including addiction to heroin, opium, and amphetamines. After every episode of amphetamine use, the patient consistently maintained a fixed kyphotic neck position for extended periods, leading to a progressive alteration in his cervical alignment. Prior to this history of addiction, there was no malalignment in his neck. During physical examination, inspection and palpation of the cervical spine revealed a pronounced kyphoscoliosis deformity. Cranial nerve testing yielded normal results. Muscle strength assessment indicated a rating of 4/5 in the upper limbs and 5/5 in the lower limbs. Upper limb paresthesia was observed, with unspecified sensory level. Deep tendon reflexes (DTRs) in the upper limbs were within normal limits, while those in the lower limbs showed a slight exaggeration. Autonomic functions were normal, and there was no evidence of sphincter dysfunction. The patient had tried various traditional and herbal remedies, but none had alleviated his symptoms. As described above, due to the severe deformity of the neck, he was admitted to our neurosurgery department at Alzahra hospital in Isfahan, Iran (Figure 1).

## 3 INVESTIGATIONS AND TREATMENT

**FIGURE 2:**
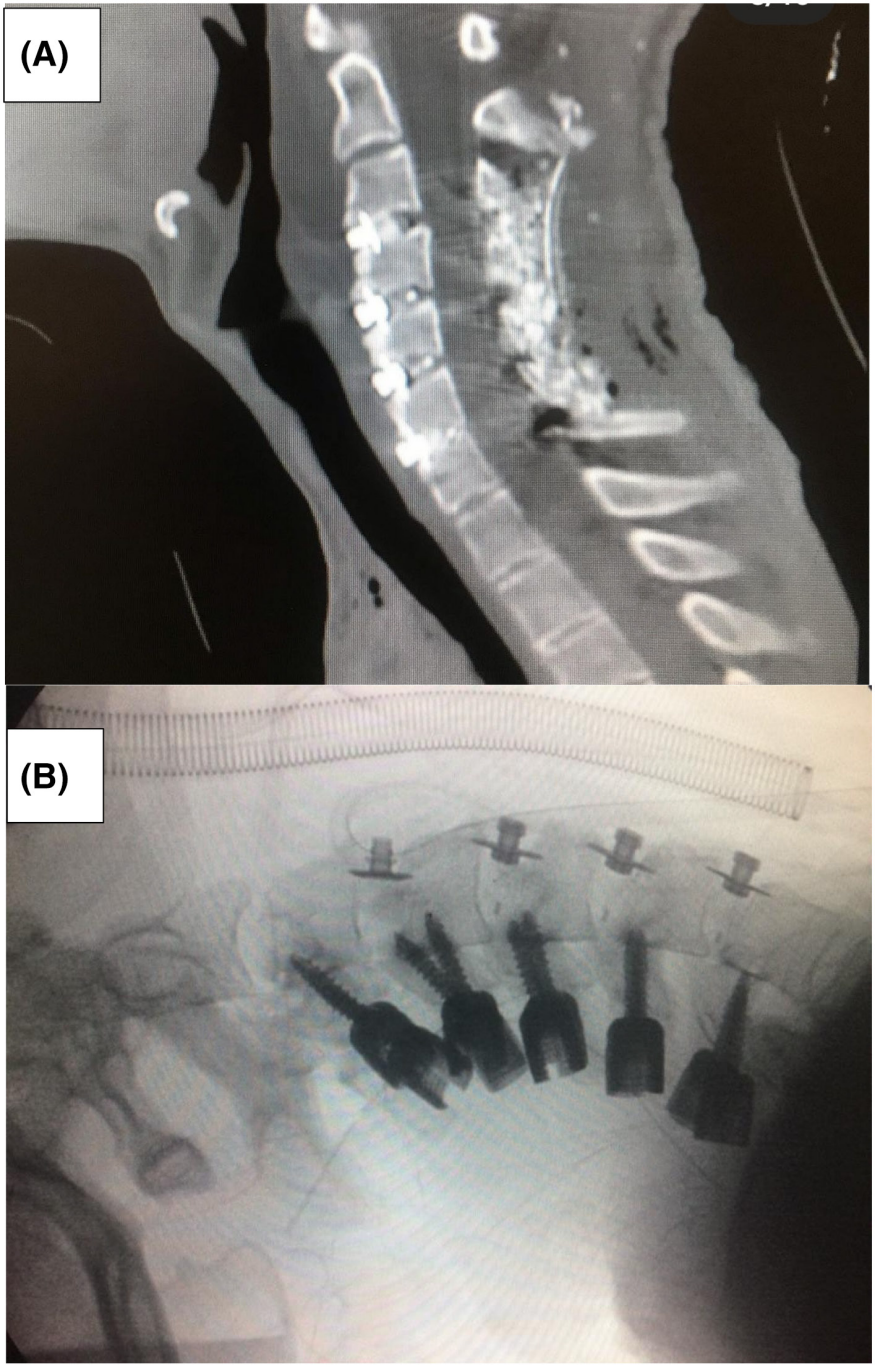
(A) Postoperative neck CT scan—sagittal view. (B) Intraoperative C‐arm fluoroscopy.

The cervical CT scan revealed a severe kyphoscoliosis deformity affecting C3, C4, and C5, accompanied by degenerative joint disease (DJD) changes in the anterior aspect of these vertebrae. No evidence of canal stenosis or fractures was observed. Subsequent MRI confirmed cervical kyphoscoliosis with unremarkable cervical cord features. Following comprehensive clinical and radiologic assessment and considering the severity of the cervical spine deformity, a decision was made to proceed with a three‐stage surgical intervention during a single anesthesia session. In addition, psychiatric and rehabilitation consultations were conducted before the surgery. Following discharge, the patient maintained scheduled appointments with the psychiatrist, having successfully discontinued drug usage. Initially, the patient was placed under general anesthesia and intubated using an endotracheal fiberoptic laryngoscope. With ongoing neuromonitoring, the patient was then carefully positioned in the prone orientation. Following the administration of anesthesia, the neck deformity became visibly reduced. Due to the inability to correct the patient's neck into the desired position, a combined surgical approach was selected. Initially, a posterior release was performed through the posterior approach, followed by a multilevel discectomy via the anterior approach. The neck was maintained in a neutral position, and a midline incision was made. Precise paraspinal muscle dissection then exposed the spinous processes, laminae, facet joints, and lateral masses bilaterally from C2 to C7. Subsequently, partial laminectomy at C3‐C4, C4‐C5, and C5‐C6, along with total bilateral facetectomy, was performed to release fibrotic adhesion. After thorough irrigation and achieving hemostasis, a drain was inserted, and the fascia and skin were carefully closed. Afterward, the patient was repositioned into the supine position. Bilateral release of the sternocleidomastoid muscles (SCMs) was performed through incisions medial to the SCM. Meticulous dissection between the carotid sheath and the tracheolaryngo‐esophago‐pharyngeal plane facilitated access to the anterior surface of the vertebral column. The longus colli muscles and fascia were dissected to expose the anterior surface of the kyphotic cervical spine. Following this, discectomy at C3‐C4, C4‐C5, C5‐C6, and C6‐C7, along with partial corpectomy at C5 and C6, was executed to reduce the kyphosis and scoliosis. Then, the placement of a locking cage in the aforementioned spaces was carried out, followed by anterior interbody fusion at C3‐C4, C4‐C5, C5‐C6, and C6‐C7 using autograft and allograft. The procedure concluded with thorough irrigation, achieving hemostasis, inserting a drain, and meticulously repairing the fascia and skin. Finally, the patient was repositioned into the prone position. The initial incision was reopened, and posterior fixation from C3 to C7 was done using lateral mass screws and rods. Posterolateral fusion at C3‐C4, C4‐C5, C5‐C6, and C6‐C7 was performed using a combination of autograft and allograft. Following irrigation, attainment of hemostasis, and drain insertion, the fascia and skin were repaired. The patient exhibited a smooth anesthetic recovery and did not experience any specific complications during the recovery period. He did not have any neurologic symptoms, and limb strength was 5/5 in all limbs. The day after surgery, the patient began to walk with the support of a hard collar which he consistently wore for the subsequent 3 months and was discharged 3 days later. A 1‐year follow‐up revealed satisfactory improvement in alignment and correction in the position of the cervical spine (Figure 2).

## 4 DISCUSSION

Kyphosis is a forward rounding spinal curvature, or bent back, resulting in a slouching or hunched posture. This condition can occur at any age, and in adults, it may be caused by tumors, degenerative spine diseases, osteoporosis, trauma, connective tissue disorders, and infections.^5^

Raj et al. documented a case of bipolar disorder treated with Haldol (haloperidol), wherein the patient developed a rigid and painful neck as an acute dystonia complication of Haldol therapy. This condition resulted in a forward flexion of the neck that remained non‐reducible even with neck extension. Ultimately, surgical intervention became necessary for the patient. This study emphasizes the association between antipsychotic drug use and the development of fixed cervical kyphosis.^6^ The primary goals of surgery for cervical spine deformities include correcting the deformity, recovering the horizontal gaze, decompressing the neural components if needed, and maintaining the surgical correction and spinal alignment with solid arthrodesis while attempting to reduce the risk of complications.^7^ The primary objectives of correcting cervical kyphosis through surgical treatment are to alleviate pain, optimize neck alignment, and mitigate or prevent neurologic deficits. Significant factors that influence the formation of the treatment plan include the location and extent of the deformity, the presence of anterior or posterior fusion, the individual's prior surgical history, and the existence of spinal cord compression.^8^ Anterior decompression is typically necessary in the presence of spinal cord compression. The flexibility of the deformity is crucial in surgical planning. In cases of fixed abnormalities, examination of facet joint fusion is required. Posterior deformity correction may be performed upon achieving the intended extension; otherwise, further release through posterior instrumentation becomes necessary. Realignment surgery is often needed for flexible deformities, while more complex, fixed deformities may require osteotomies or a combination of an anterior–posterior approach. The location of the deformity is critical; both anterior corpectomy and fusion are frequently used in treating focal kyphosis of the cervical spine.^9^ Cervical kyphosis can be surgically treated through circumferential or pedicular subtraction osteotomies using anterior, posterior, or combination methods. For the most effective outcome, posterior instrumented fusion should be employed in cases of anterior corpectomy and fusion, especially for post‐laminectomy kyphosis.^10^ In our case, the criteria for this syndrome include a dropped head for an extended period, leading to severe cervical kyphoscoliosis that cannot be better explained by any other reason. We discovered that drug abuse contributed to the uncommon development of severe complicated cervical kyphosis. The drug does not have a direct effect on musculoskeletal changes. Instead, there is an indirect effect: When the patient uses the drug, they remain in a certain position for a long time, and over months, this results in musculoskeletal changes that lead to kyphoscoliosis. Even with vigorous neck extension, the significant kyphosis left very little room between the chin and the manubrium of the sternum. We planned a three‐stage operation, commencing with a posterior approach, followed by an anterior approach, and concluding with another posterior approach. During the initial posterior approach, laminectomies were performed to achieve posterior decompression of the spinal cord. This crucial step created the necessary space and ensured the safety of the cord for subsequent phases of the operation. Additionally, bilateral total facetectomies were executed to release fibrotic adhesions. The subsequent anterior approach involved discectomy and partial corpectomy, coupled with the placement of locks to achieve partial correction of the kyphosis. Ultimately, the cervical spine was stabilized in the appropriate position through posterior fixation in the last phase of the operation.

## 5 CONCLUSION

In this case report, we presented a case of DHS that deviates from established etiologic norms. Remarkably, the patient lacked any pertinent history of cervical trauma, underlying diseases, or indications of infection based on thorough physical examinations and laboratory tests. Our meticulous investigation led us to a distinctive conclusion: the onset of this condition following the use of heroin and amphetamine can attribute to intoxication. In light of this novel insight, we have designated this particular manifestation as “intoxicated syndrome.” This pioneering nomenclature underscores the imperative connection between drug abuse and the manifestation of DHS, along with accompanying symptoms such as decreased limb force and paresthesia. To advance clinical understanding and management of similar cases, we propose the term “intoxicated syndrome” for patients presenting with this unique constellation of symptoms resulting from substance abuse. Moreover, based on our findings, we advocate for a structured three‐stage surgical intervention as an optimal approach to similar DHS cases for addressing the complexities associated with intoxicated syndrome. This proposed treatment paradigm aims to provide a comprehensive and effective strategy for managing this distinct subset of patients, thus contributing to the evolving landscape of medical knowledge and care in this field.

## AUTHOR CONTRIBUTIONS

Majid Rezvani: Conceptualization, methodology, project administration, visualization, and writing–review and editing. Seyedali Modarres Sadeghi: Conceptualization, resources, writing–original draft, and writing–review and editing. Farid Masaeli: Resources, writing–original draft, and writing–review and editing. Anish Thapa: Conceptualization, writing–original draft, and writing–review and editing. Ashani Shah: Resources, writing–original draft, and writing–review and editing. Farhad Mahmoudi: Conceptualization, investigation, methodology, project administration, resources, supervision, visualization, writing–original draft, and writing–review and editing.

## FUNDING INFORMATION

No funding was received for this article.

## CONFLICT OF INTEREST STATEMENT

The authors have no conflicts of interest to declare.

## ETHICS STATEMENT

The patient has been de‐identified. Any images used do not permit the identification of the individual. Otherwise, there are no ethical concerns in this manuscript. There was no ethics approval required for this manuscript.

## CONSENT

Written informed consent was obtained from the patient to publish this report in accordance with the journal's patient consent policy.

## Data Availability

The data that support the findings of this study are available from the corresponding author upon reasonable request.
